# Genotypic and Phenotypic Characterization of Clinical *Escherichia coli* Sequence Type 405 Carrying IncN2 Plasmid Harboring *bla*_NDM-1_

**DOI:** 10.3389/fmicb.2019.00788

**Published:** 2019-04-12

**Authors:** Yingying Hao, Chunhong Shao, Xu Geng, Yuanyuan Bai, Yan Jin, Zhiming Lu

**Affiliations:** Department of Clinical Laboratory, Shandong Provincial Hospital Affiliated to Shandong University, Jinan, China

**Keywords:** *bla*_NDM-1_, ST405, *Escherichia coli*, IncN2, China, carbapenem-resistant Enterobacteriaceae

## Abstract

We report a *bla*_NDM_-carrying ST405 *Escherichia coli* recovered from the abdominal fluid of a patient in Shandong, China. This strain belonged to the high-risk phylogenetic group D and carried the virulence genes, *papG II, papG III, papC*, and iroN. In addition to *bla*_NDM-1_, this isolate carried the quinolone resistance gene *acc(6′)-Ib* and extended-spectrum β-lactamase (ESBL) genes *bla*_CTX-M-15_ and *bla*_CTX-M-14_. *bla*_NDM-1_ was located on a 41 Kb IncN2 self-transmissible plasmid. The IncN2 plasmid named as pJN24NDM1 was fully sequenced and analyzed. Genome comparative analysis showed that IncN2 plasmids harboring carbapenem-resistance genes possessed conserved backbones and variable accessory regions. Phylogenetic analysis of 87 IncN plasmids based on orthologous genes indicated that 9 IncN2 plasmids fell into one phylogenetic clade, while 4 IncN3 plasmids were in two phylogenetic clades of the 74 IncN1 plasmids. The presence of IncN2 plasmids harboring *bla*_NDM_ in the high-risk clone ST405 *E. coli* raises serious concerns for its potential of dissemination.

## Introduction

New Delhi metallo-beta lactamase (NDM)-producing Enterobacteriaceae have emerged as a critical public-health problem worldwide (Ishii et al., 2017; Livorsi et al., 2018). Recently, a study from the French National Reference Center showed that 21% of 140 carbapenem-resistant isolates produced NDM carbapenemase (Gauthier et al., 2018). Nationwide surveillance of clinical carbapenem-resistant Enterobacteriaceae in China showed that NDM production was predominantly associated with carbapenem resistance in *Escherichia coli* (Zhang et al., 2017; Li et al., 2018; Wang et al., 2018). In other countries such as India, Pakistan, and the Balkan countries, infections caused by NDM-producer has created large medical and economic burdens (Otter et al., 2017). Notably, *bla*_NDM_-carrying strains have also been isolated from pets, food animals, and environmental samples such as sewage water and urban rivers (Hao et al., 2018). The presence of NDM-producer in the community creates an additional risk of dissemination by travelers (Zhong et al., 2017). Targeted resistance surveillance and interventions are needed to control the expanding global distribution of *bla*_NDM_-carrying strains.

Extraintestinal pathogenic *E. coli* (ExPEC) is a leading cause of severe infections and has become increasingly resistant to broader classes of antimicrobial agents (Peirano et al., 2013). NDM-producing ST405, phylogenetic group D *E. coli* is an emerging ExPEC pathogen with sporadic reports from Canada, Denmark, Spain, the United Kingdom, Pakistan, Italy, India, and China (Jakobsen et al., 2014; Peirano et al., 2014; Toleman et al., 2015; Ranjan et al., 2016; Barrado et al., 2018; Qamar et al., 2018; Zhang et al., 2018). *bla*_NDM_-carrying IncB/O, IncFII, IncFIA, and IncFIb type plasmids have been identified in ST405 *E. coli* strains.

In this study, we report a ST405 *E. coli* isolate producing *bla*_NDM-1_ harbored by an IncN2 plasmid recovered from a patient with intra-abdominal infection in our hospital. This is the first report of the *bla*_NDM_-harboring IncN2 plasmid carried by ST405 *E. coli*. There are only 8 fully sequenced IncN2 plasmids harboring carbapenemase in the National Center for Biotechnology Information (NCBI) database. The molecular characteristics of the IncN2 plasmid harboring *bla*_NDM_ were determined by genomic comparison and phylogenetic analyses.

## Materials and Methods

### Clinical Case, Clinical Strain, and Susceptibility Assays

The carbapenem-resistant *E. coli* strain JN24 was isolated from a 58-year old man with abdominal infection who had been admitted to a teaching hospital in Shandong, China in 2015. Pancreaticoduodenectomy was conducted for the patient to treat distal bile duct carcinoma. Abdominal infection and a persistent high temperature of 39.5°C was observed at 1 week after surgery. The patient was administered intravenously with imipenem and vancomycin, but he failed to respond to the treatment. His condition deteriorated, and he quit therapy in the next week because of the financial burden of the treatment. *E. coli* JN24 was recovered from the abdominal fluid of this patient. Although blood culture was not performed, bacteremia was highly suspected. The patient had no history of travel. His medication history and hospital course were obtained from the hospital information system. The case history collection and report were approved by the Ethics Committee of Shandong Provincial Hospital. Species identification of strain JN24 was conducted with the VITEK2 automated system (BioMérieux, Marcy-l’Étoile, France) in the clinical microbiology laboratory of Shandong Provincial Hospital. The carbapenem inactivation method (CIM) and EDTA-modified CIM method were performed for phenotypic detection of carbapenemases.

Minimum inhibitory concentration (MIC) testing was conducted using *E*-test Strips for aztreonam, cefepime, cefotaxime, cefoxitin, ceftazidime, ertapenem, imipenem, meropenem, piperacillin-tazobactam, trimethoprim-sulfamethoxazole, ciprofloxacin, gentamicin, amikacin, polymyxin B, fosfomycin, and tigecycline. The MIC results were interpreted using Clinical Laboratory Standards Institute criteria (CLSI, 2017), except for polymyxin B and fosfomycin, which were interpreted using the EUCAST criteria (EUCAST, 2017).

### Molecular Typing and Phylogenetic Group Genotyping

Multilocus sequence typing (MLST) of the isolate was conducted using a standard method according to the guidelines on the *E. coli* MLST website (Bitar et al., 2017). The phylogenetic group of JN24 was determined by a multiplex PCR-based method using the primers *chuA, yjaA*, and *TspE4.C2* (Wirth et al., 2006). Multiplex PCR assays were conducted to detect virulence-associated genes as described previously (Wang L. H. et al., 2016).

### Molecular Detection of Resistance Genes

Antimicrobial resistance genes were detected by PCR as described previously (Zhu et al., 2016). These antimicrobial resistance genes included carbapenemase-encoding genes, extended-spectrum β-lactamase genes, plasmid-mediated pAmpC β-lactamase, 16S rRNA methylase genes, plasmid-mediated fosfomycin resistance genes, plasmid-mediated quinolone resistance genes, and polymyxin B resistance genes (mcr-1), which have been described previously (Du et al., 2016; Zhu et al., 2016).

### *bla*_NDM_-Carrying Plasmid Analysis and Sequencing

Plasmid conjugation experiments were performed as described previously using E. coli J53Azi^R^ as the recipient and strain JN24 as the donor. Transconjugants were screened on Mueller-Hinton agar plates with sodium azide (100 μg/mL) and ceftazidime (6 μg/mL). The transconjugant J24 and recipient J53AziR were subjected to antimicrobial susceptibility testing using a method similar to that used for the clinical strain.

S1 pulsed field gel electrophoresis (S1-PFGE) was conducted to evaluate the size and amounts of plasmids carried by the clinical strain JN24 and transconjugant J24. Southern blotting was performed to determine the location of *bla*_NDM-1_. The genomic DNA of the clinical strain and transconjugant embedded in the gel plugs was digested with QuickCut S1 nuclease (Takara, Shiga, Japan) for 1 h, and then separated by PFGE for 17 h with the pulse time switched from 2.16 to 63.8 s. *Salmonella* strain H9812 was digested with QuickCut *Xba*I (Takara, Shiga, Japan) and used as the reference marker (Liu et al., 2018). Plasmid DNA was transferred to a nylon membrane and analyzed by Southern blotting using specific *bla*_NDM_ probes labeled with digoxigenin (Roche, Basel, Switzerland) (Liu et al., 2018).

We tried to eliminate pJN24NDM1 from the clinical strain JN24 to evaluate the antibiotic resistance profile of JN24 without the NDM-plasmid. A culture of the clinical isolate JN24 was grown overnight at 37°C on a Luria-Bertani agar plate, and then transferred to a fresh Luria-Bertani agar plate. After repeated passage for 10 days, 10 colonies were randomly selected and screened for *bla*_NDM_ by PCR (Zhu et al., 2016).

Genomic DNA of the plasmid carrying *bla*_NDM_ was prepared using the Wizard^®^ Genomic DNA Purification Kit (Promega, Madison, WI, United States) and sequenced with 150× coverage using an Illumina HiSeq X Ten (San Diego, CA, United States) at MajorBio Co. (Shanghai, China). Reads were trimmed and assembled using SOAPdenovo2. PCR and Sanger sequencing were conducted at Sangon Biotech (Shanghai, China) to close the gaps. The plasmid sequence was screened for resistance genes using the Comprehensive Antibiotic Resistance Database^1^ and the plasmid type was established using PlasmidFinder at the Center for Genomics and Epidemiology^2^. Coding sequence prediction of the plasmid was performed with Rapid Annotations using Subsystems Technology and annotation of the predicted open reading frames was conducted by blast against the non-redundant protein database. Mobile elements were detected using ISFinder and the Tn Number Registry.

### Genome Comparisons

For comparative analysis, 86 plasmids harboring carbapenemases with IncN1, IncN2, or IncN3 type replicons were downloaded from the NCBI. The presence of carbapenemases was screened by blasting against the Comprehensive Antibiotics Resistance database. Comparative analysis of the genetic contexts among IncN plasmids was conducted using a Large-Scale Blast Score Ratio (LS-BSR) pipeline (Hazen et al., 2018) and displayed in a pheatmap. Eight fully sequenced IncN2 plasmids harboring carbapenemases were compared to pJN24NDM1 by BLAST Ring Image Generator (Liu et al., 2018). More detailed genome alignment was conducted by local BLAST and visualized with Easyfig (Zhu et al., 2016).

### Phylogenetic Analysis of IncN Plasmids Harboring Carbapenemases

Among the 87 plasmids, homologs were identified using the OrthoFinder and a set of 56 genes representing the core genome was summed. Orthologous genes were aligned by Multiple Alignment using Fast Fourier Transform, and the maximum-likelihood phylogenetic tree was inferred using RAxML with a 1000-bootstrap test (Long et al., 2019).

## Results and Discussion

### Strain Features

The antibiotic MICs of *E. coli* strain JN24 are listed in Table 1. The strain was resistant to quinolones, trimethoprim-sulfamethoxazole, piperacillin-tazobactam, carbapenems, aztreonam, and cephalosporins but remained susceptible to polymyxin B, aminoglycosides, fosfomycin, and tigecycline. The isolate was established as a metallo-beta-lactamase (MBL)-producer by CIM and EDTA-modified CIM. In addition to *bla*_NDM-1_, the strain was found to carry *bla*_CTX-M-15_, *bla*_CTX-M-14_, and the plasmid-mediated quinolone resistance gene *acc(6′)-Ib*. Therefore, this strain was highly resistant to β-lactam drugs and showed multidrug resistance. Plasmid-mediated pAmpC β-lactamase, fosfomycin resistance genes, and the colistin B resistance gene were not detected.

**Table 1:** Antimicrobial resistance profile of *Escherichia coli* JN24 and the transconjugant J24.

MIC (μg/mL)																	
isolates	CZO	CRO	CAZ	FOX	FEP	ATM	TZP	MEM	ETP	IMP	SXT	AK	CN	CIP	LEV	FOS	TGC
J53Azi^R^	<=0.016	<=0.016	<=0.016	<=0.016	<=0.016	<=0.016	<=0.016	<=0.02	<=0.02	<=0.02	<=0.02	<=0.016	<=0.016	<=0.02	<=0.02	2	0.38
J24	>=256	>=256	>=256	>=256	16	0.64	>=256	8	12	16	0.32	1	0.5	<=0.02	<=0.02	2	0.38
JN24	>=256	>=256	>=256	>=256	>=256	>=256	>=256	>=32	>=32	>=32	>=32	>=256	>=256	>=32	>=32	2	0.38

*MIC, minimal inhibitory concentrations; CZO, cefazolin; CRO, cefatriaxone; CAZ, ceftazidime; FOX, cefoxitin; FEP, cefepime; ATM, aztreonam; TZP, piperacillin/tazobactam; MEM, meropenem; ETP, ertapenem; IPM, imipenem; STX, trimethoprim/sulfamethoxazole; AK, amikacin; CN, gentamicin; CIP, ciprfloxacin; LEV, Levofloxacin; FOS, Fosfomycin; TGC, tigecycline.*

The clinical *E. coli* strain JN24 was assigned to the high-risk clone ST405 and phylogenetic group D. This strain carried multiple virulence genes, including *kpsMT III* type *cps, papG II, papG III, papC*, and *iroN*, which enhanced its adhesion, colonization, and Fe availability. According to a multicenter study of *E. coli* bloodstream infections in Shanghai, ST131 belonging to phylogroup B2 and ST405 belonging to phylogroup D contributed briefly to the dissemination of EC-BSI in Shanghai (Wang S. et al., 2016). Notably, 3 of the 6 carbapenem-resistant isolates were D-ST405 *E. coli*. Other studies also reported that ST405 *E. coli* isolates harboring CTX-M may be related to NDM β-lactamase production (Wang S. et al., 2016). Additional studies focused on the reliable molecular surveillance of high-risk clones with MDR are needed to prevent their further dissemination.

### Characterization of *bla*_NDM_-Harboring Plasmid

The plasmid pJN24NDM1 from strain JN24 harboring the New Delhi metallo-beta-lactamase was successfully transferred into *E. coli* J53Azi^R^ by conjugation. The presence of *bla*_NDM-1_ and absence of *bla*_CTX-M-15_, *bla*_CTX-M-14_, and *acc(6′)-Ib* in the transconjugant J24 was confirmed by PCR. The transconjugant was resistant to carbapenems and cephalosporin but was susceptible to quinolones, aminoglycosides, and aztreonam. S1-PFGE and Southern blot analysis revealed that the clinical strain JN24 carried two plasmids. *bla*_NDM_ was located on the plasmid and showed a size of approximately 41 kb (Supplementary Figure S1). We failed to eliminate the plasmid harboring *bla*_NDM_ from the clinical isolate JN24, indicating that this plasmid was stable in isolate JN24.

The complete plasmid sequence was determined to better characterize the plasmid pJN24NDM1 harboring *bla*_NDM-1_. pJN24NDM1 is a 41,190-base pair (bp) plasmid that belongs to the IncN2 incompatibility group according to replication module analysis. In pJN24NDM1, the genetic environment of *bla*_NDM_ in pJN24NDM1 was MITE-*ΔtnpA-ISEc33-bla_NDM_-bleMBL-ΔtnpF-ISSen4-ΔaphA6-Tn5403* and that in IncX3 was *IS3000-ISAba125-IS5-bla_NDM_-bleMBL-trpF-dsbC-IS26-ΔumuD*. *bla*_NDM-1_ was embedded by a miniature inverted repeat element (MITE) and *ISAba125* was interrupted by *ISEc33* in the upstream sequence, followed by *ble-ISSen4*-*Tn5403* in the downstream region. The insertion of Tn125-like elements by MITE in IncN2 plasmids indicated mobilization of *bla*_NDM-1_. The *Tn125*-like element in pJN24NDM1 lacked the fragments of *dsbC, cutA, groES, groEL*, and *ISCR27*, which are present in the classical *Tn125* element (Jiang et al., 2017; Hao et al., 2018).

### Comparative Analysis of IncN2 Plasmids Harboring Carbapenemases

**FIGURE 1:**
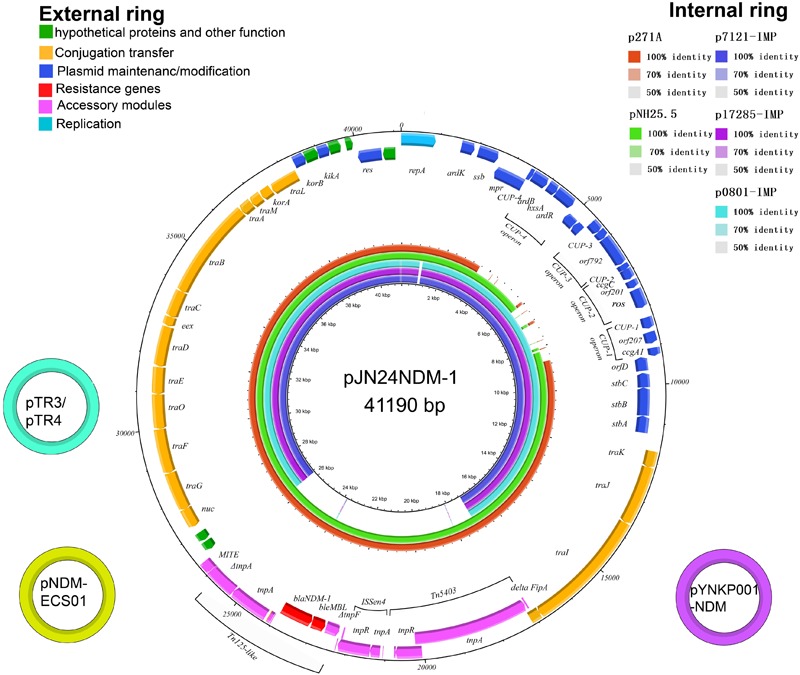
The external ring is the schematic representation of plasmid pJN24NDM1 (GenBank accession no. MK368725). The three small external rings display the IncN2 type plasmids harboring *bla*_NDM-1_ with identity >99%. The genes with different functional annotations were labeled with different colors. The internal five rings showed the comparative analysis of *bla*_NDM_-harboring IncN2 plasmids with pJN24NDM1, including p7121-IMP (purple), p17285-IMP (violet), p0801-IMP (sky blue), p271A (orange), and pNH25.5 (green) (constructed by BRIG).

Eight fully sequenced IncN2 plasmids harboring carbapenemases obtained from the NCBI database were compared to pJN24NDM1, including pTR3 (GenBank accession no. JQ349086), pNDM-ECS01 (GenBank accession no. KJ413946), pYNKP01-NDM (GenBank accession no. KP900017), p271A (GenBank accession no. JF785549), pNH25.5 (GenBank accession no. NZ_CP024879), p7121-IMP (GenBank accession no. KX784502), p17285-IMP (GenBank accession no. KX784503), and p0801-IMP (GenBank accession no. KT345947) (Figure 1; Poirel et al., 2011; Chen et al., 2012; Netikul et al., 2014; Jiang et al., 2017).

Sequence alignment by BRIG showed that pJN24NDM1 was identical to the previously reported plasmid pNDM-ECS01 from ST131 *E. coli* isolated from Thailand and China and the plasmid pYNKP01-NDM from *Raoultella ornithinolytica* isolated from Mainland China (Netikul et al., 2014; Sun et al., 2015). Additionally, the pJN24NDM1 plasmid showed 99% nucleotide identity with plasmid pTR3 of *Klebsiella pneumoniae* isolated from Singapore (Chen et al., 2012). These three locations are geographically contiguous, but a history of travel was denied by our patient. Thus, the IncN2 plasmids may have exit in these locations naturally or are highly transmissible. pJN24NDM1 was similar to the plasmids p17285-IMP, p7121-IMP, and p0801-IMP isolated from China, but were in different regions and the NDM cassette was replaced with the IMP cassette and a putative iteron of repA (Figure 1).

**FIGURE 2:**
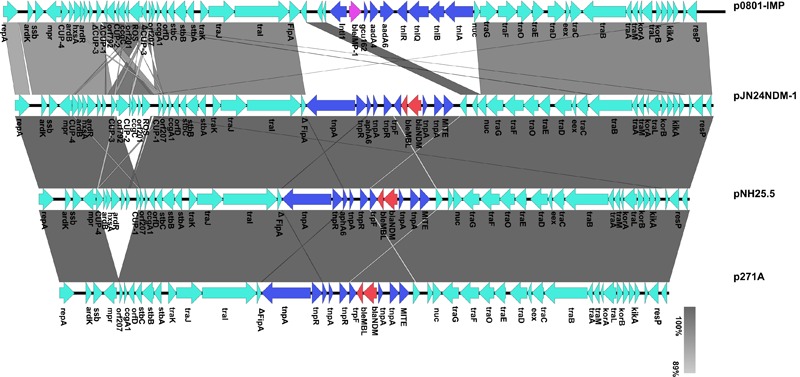
Comparison of genetic context of the IncN2 plasmids harboring *bla*_NDM_ with pJN24NDM1.

To determine the detailed structural differences between the plasmids pJN24NDM1, p271A, pNH25.5, pYNKP01-NDM, and p0801-IMP, additional linear comparative genomics analysis of these four plasmids was performed by BLAST. The plasmids showed similar genomic contents and varied mainly at the conserved upstream repeat (CUP) region and accessory region. Compared to pJN24NDM1, p271A lacked a 5,243-bp CUP-controlled regulon region, possibly because of recombination between CUP sequences. Deletion of CUP-2 and CUP-3 was also observed in pNH25.5. In p0801-IMP, CUP-3 and CUP-1 were disrupted and recombined, leading to inversion of *orf*792 to the ROS region. Compared to pJN24NDM1, the accessory region harboring a resistance gene was replaced with a class 1 integron *In1223* in p0801-IMP (19) (Figure 2).

### Genomic and Phylogenetic Analysis

The 86 IncN plasmids carrying carbapenemases downloaded from the NCBI database were sub-grouped into three types according to their replication genes: IncN1 (*n* = 74), IncN2 (*n* = 8), and IncN3 (*n* = 4) (Supplementary Table S1; Kim et al., 2016). Multiple carbapenemases were detected in these plasmids including IMP, VIM, NDM, KPC, and OXA.

**FIGURE 3:**
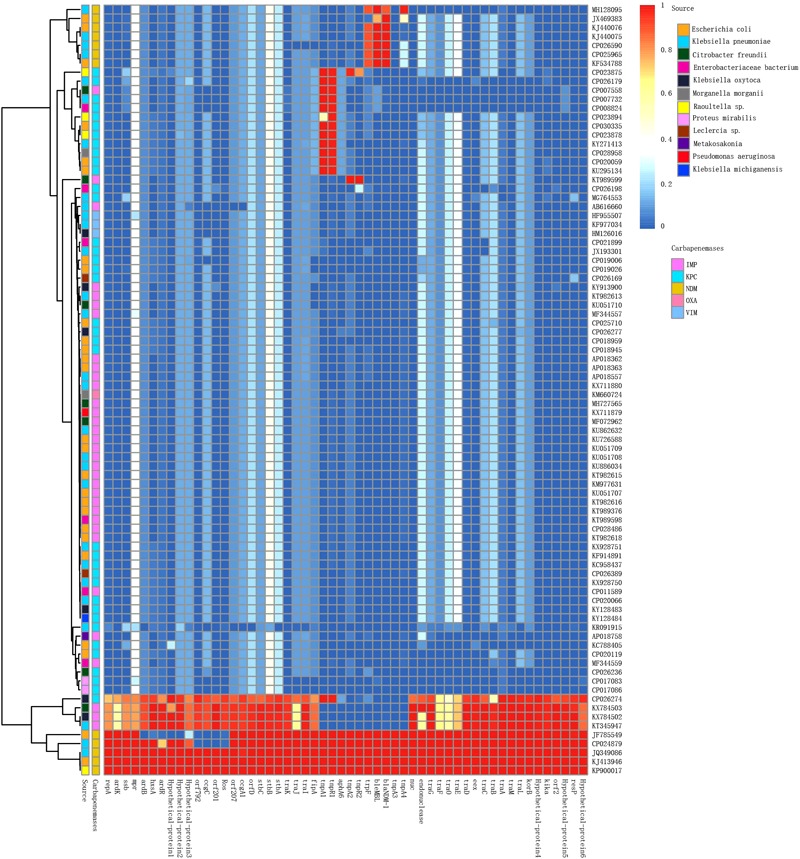
Detection of plasmid pJN24NDM1 genes in the IncN plasmids harboring carbapenemases analyzed in this study. The BLAST score ratio (BSR) values in the heatmap represented the existence (red) or non-existence (blue) of gene of the plasmid pJN24NDM1 in each of the plasmids. Rows represented each of the CDS of pJN24NDM1, while the column represented a different palsmid. The source and carbapenemase of each plasmids were marked by squares at the top of the heat map. The detailed information of the plasmids was listed in the Supplementary Table S1.

IncN plasmids have been reported globally but are mainly prevalent in China and the United States. Of the 38 IncN1 plasmids reported in China (including the mainland and Taiwan, China), the major carbapenemase type was *bla*_IMP_ (63.9%; 23/36), followed by *bla*_NDM_ (19.4%; 7/36), and *bla*_KPC_ (19.4%; 7/36). Of the 25 IncN1 plasmids reported in the United States, the major carbapenemase type was *bla*_KPC_, which accounts for 88% (22/25) of IncN1 plasmids. Seven of the eight IncN2 plasmids were reported in Asia and are mainly prevalent in China (6/8). The plasmid p271A isolated from a patient in Australia was transferred from Bangladesh in South Asia (Figure 3).

IncN plasmids were detected in 16 types of bacteria, with *E. coli* and *K. pneumoniae* as the most frequent reservoirs. Notably, P378-IMP harboring *bla*_NDM_ was recovered from *Pseudomonas aeruginosa*, which is an important non-fermentative bacterium causing hospital-acquired infections. The recombinogenic capability may endow broad host plasmids to spread multidrug resistance to other bacteria species.

**FIGURE 4:**
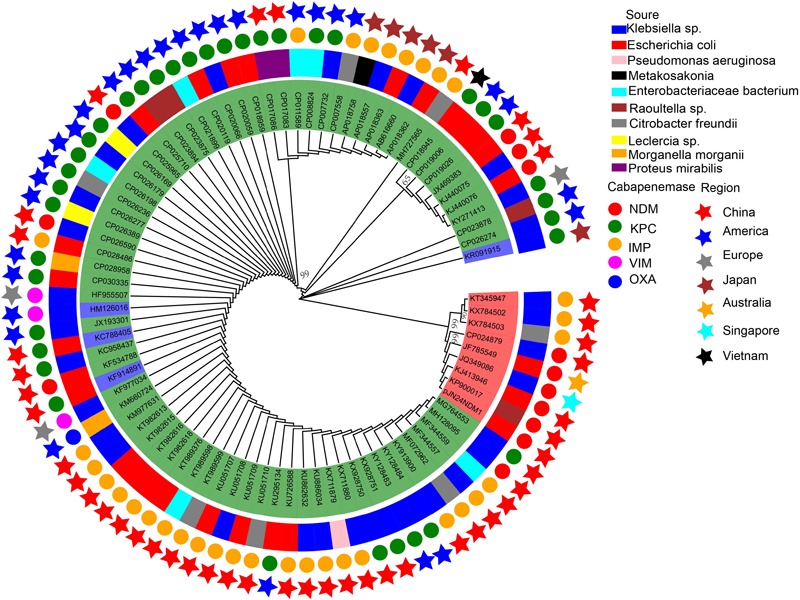
Phylogenetic analysis of IncN plasmids harboring carbapenemases.

To compare the genetic contents of three subtype IncN plasmids, the LS-BSR pipeline was used. pNDM-ECS01 was used as the reference genome for LS-BSR, considering that the 5243 bp within the CUP controlling the regulon in p271A (first sequenced IncN2 plasmid) was absent. The collection of IncN2 plasmids showed a backbone arrangement similar to the IncN1 and IncN3 collection, while nucleotide sequence homology showed limited similarity (Figure 3). According to maximum-likelihood phylogenetic analysis, the IncN2 plasmids were in one phylogenetic clade, while 4 IncN3 plasmids were in 2 phylogenetic clades of 74 IncN1 plasmids (Figure 4).

## Conclusion

In this study, a self-transmissible IncN2 plasmid bearing *bla*_NDM_ was detected in ST405 *E. coli*, which is a globally disseminated lineage. We highlight the dissemination potential of IncN2 plasmids harboring *bla*_NDM_. The presence of *bla*_NDM_ mediated by plasmids in the high-risk clone ST405 *E. coli* linked with multiple virulence genes and resistance genes is increasing, which may create health concerns.

## Author Contributions

YJ and ZL designed the experiments. YH analyzed the data and wrote the manuscript. CS and XG prepared the table and figures. YB carried out the bacteria identification.

## Conflict of Interest Statement

The authors declare that the research was conducted in the absence of any commercial or financial relationships that could be construed as a potential conflict of interest.

## Footnotes

1 https://card.mcmaster.ca/

2 https://cge.cbs.dtu.dk/services/

## Supplementary Material

FIGURE S1 S1-PFGE and Southern Blot patterns of clinical strain JN24 **(A)** and transconjugant J24 **(B)** M, marker; *Salmonella enterica* serotype H9812.

TABLE S1 The plasmids analyzed in the large-scale BLAST score ratio analysis and Phylogenetic Analysis.
