# Supplementary figures and images for: Genotypic and Phenotypic Characterization of Clinical Escherichia coli Sequence Type 405 Carrying IncN2 Plasmid Harboring blaNDM-1
Source: Front Microbiol. 2019 Apr 12;10:788. doi: 10.3389/fmicb.2019.00788 (PMC6499153; doi:10.3389/fmicb.2019.00788)

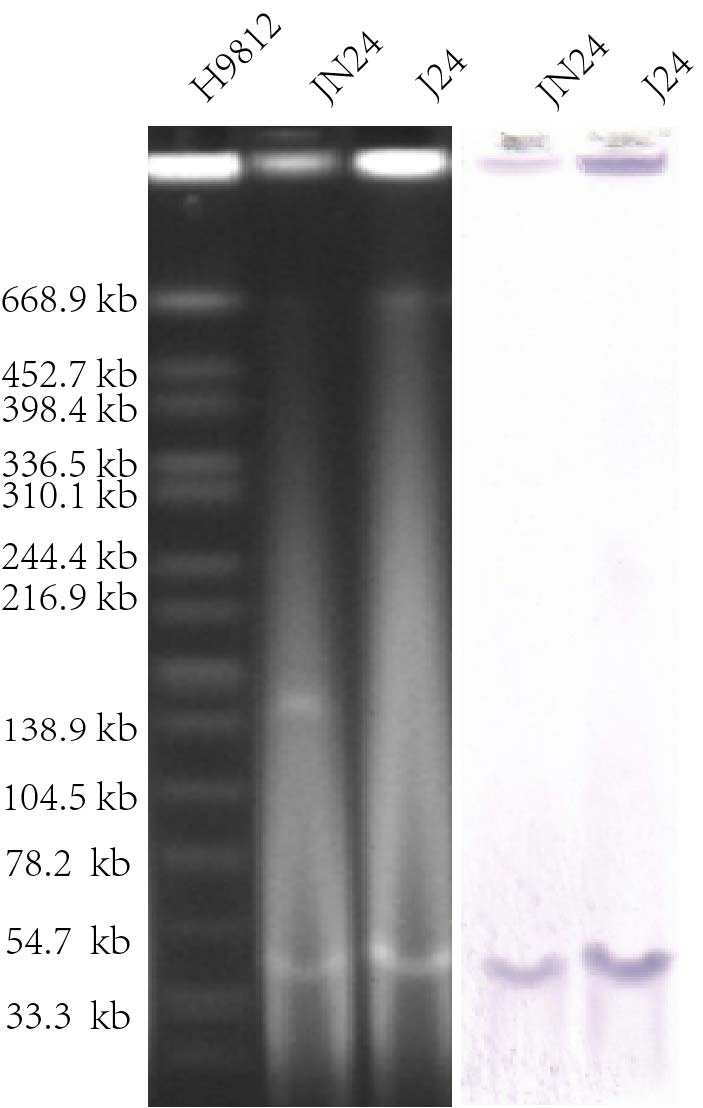

Supplement: FIGURE S1 — S1-PFGE and Southern Blot patterns of clinical strain JN24 (A) and transconjugant J24 (B) M, marker; Salmonella enterica serotype H9812. [file Image_1.JPEG]
