# Supplementary material for: Genotypic and Phenotypic Characterization of Clinical Escherichia coli Sequence Type 405 Carrying IncN2 Plasmid Harboring blaNDM-1
Source: Front Microbiol. 2019 Apr 12;10:788. doi: 10.3389/fmicb.2019.00788 (PMC6499153; doi:10.3389/fmicb.2019.00788)
Supplement: TABLE S1 — The plasmids analyzed in the large-scale BLAST score ratio analysis and Phylogenetic Analysis. [file Table_1.DOCX]

| **Accession NO.** | **Plasmid** | **Carbapenemase** | **Source** | **Length(bp)** |
| --- | --- | --- | --- | --- |
| AB616660 | pKPI-6 | IMP-6 | Klebsiella Pneumoniae | 47236 |
| AP018362 | pA56-1S | IMP-6 | Escherichia coli | 53198 |
| AP018363 | pA56-1R | IMP-6 | Escherichia coli | 53989 |
| AP018557 | pMTY14343_IncN | IMP-1 | Klebsiella Pneumoniae | 51787 |
| AP018758 | pMRY16-398_2 | IMP-6 | Metakosakonia | 47417 |
| CP007558 | pKEC-a3c | IMP-6 | Citrobacter freundii | 272297 |
| CP007732 | pKEC-dc3 | KPC-2 | Klebsiella Pneumoniae | 268334 |
| CP008824 | pKEC-39c | KPC-1 | Enterobacter cloacae | 319976 |
| CP011589 | pKPC_CAV1043 | IMP-6 | Enterobacter asburiae | 59138 |
| CP017083 | pT211 | KPC-1 | Proteus mirabilis | 24225 |
| CP017086 | pT18 | KPC-1 | Proteus mirabilis | 59035 |
| CP018945 | pEC224_KPC | KPC-1 | Escherichia coli | 55436 |
| CP018959 | pEC422_KPC | KPC-1 | Escherichia coli | 51885 |
| CP019006 | pECAZ159_2 | KPC-2 | Escherichia coli | 47531 |
| CP019026 | pEC881_KPC | KPC-1 | Escherichia coli | 59373 |
| CP020059 | AR_0061 | KPC-3 | Escherichia coli | 87982 |
| CP020066 | AR_0117 | KPC-3 | Klebsiella Pneumoniae | 72663 |
| CP020119 | AR_0104 | KPC-4 | Escherichia coli | 51515 |
| CP021899 | AR_0050 | KPC-4 | Enterobacter cloacae | 69635 |
| CP023875 | FDAARGOS_429 | KPC-3 | Raoultella planticola | 262185 |
| CP023878 | FDAARGOS_430 | KPC-3 | Raoultella planticola | 108606 |
| CP023894 | FDAARGOS_431 | KPC-3 | Raoultella ornithinolytica | 71267 |
| CP025710 | pYDC107_70 | KPC-3 | Escherichia coli | 70372 |
| CP025965 | pNDM1_LL34 | NDM-1 | Klebsiella Pneumoniae | 59730 |
| CP026169 | pKPC-79f0 | KPC-1 | LEnterobacter cloacaeercia sp | 58627 |
| CP026179 | pKPC-224e | KPC-1 | Klebsiella Pneumoniae | 237571 |
| CP026198 | pKPC-c606 | KPC-1 | Enterobacteriaceae bacterium | 174695 |
| CP026236 | pKPC-59e4 | KPC-1 | Citrobacter freundii | 65291 |
| CP026274 | pKPC-4b66 | KPC-1 | Klebsiella oxytoca | 235655 |
| CP026277 | pKPC-8bc0 | KPC-1 | Klebsiella oxytoca | 56561 |
| CP026389 | pKPC-3714 | KPC-1 | LEnterobacter cloacaeercia sp. | 50333 |
| CP026590 | p4 | NDM-1 | Klebsiella Pneumoniae | 49215 |
| CP028486 | p3 | IMP-4 | Escherichia coli | 52864 |
| CP028958 | AR_0133 | KPC-1 | Morganella morganii | 71304 |
| CP030335 | AR_451 | KPC-3 | Escherichia coli | 75281 |
| HF955507 | pK45-67VIM | VIM-1 | Klebsiella Pneumoniae | 56171 |
| JX193301 | pBK31551 | KPC-4 | Klebsiella Pneumoniae | 83712 |
| JX469383 | pEcNDM1 | NDM-1 | Escherichia coli | 58228 |
| KC958437 | pKo6 | KPC-1 | Klebsiella Pneumoniae | 65549 |
| KF534788 | pNDM-BTR | NDM-1 | Escherichia coli | 59400 |
| KF977034 | pOW16C2 | VIM-1 | Klebsiella Pneumoniae | 59228 |
| KJ440075 | pLK78 | NDM-1 | Klebsiella Pneumoniae | 56072 |
| KJ440076 | pLK75 | NDM-1 | Escherichia coli | 56489 |
| KM660724 | pMR3-OXA181 | OXA-181 | Morganella morganii | 57797 |
| KM977631 | pIMP-1495 | IMP-4 | Klebsiella Pneumoniae | 50742 |
| KT982613 | pIMP1496 | IMP-4 | Klebsiella Pneumoniae | 50979 |
| KT982615 | pIMP-FS1505 | IMP-4 | Escherichia coli | 54449 |
| KT982616 | pIMP-HK1509 | IMP-4 | Escherichia coli | 54449 |
| KT982618 | pIMP-GZ1517 | IMP-4 | Escherichia coli | 51589 |
| KT989376 | pIMP-SZ1515 | IMP-4 | Escherichia coli | 54449 |
| KT989598 | pIMP-SH1506 | IMP-4 | Enterobacter cloacae | 54669 |
| KT989599 | pIMP-HK1500 | IMP-4 | Citrobacter freundii | 53653 |
| KU051707 | pIMP-SZ1502 | IMP-4 | Escherichia coli | 51362 |
| KU051708 | pIMP-SZ1501 | IMP-4 | Klebsiella Pneumoniae | 51469 |
| KU051709 | pIMP-GZ1058 | IMP-4 | Escherichia coli | 51600 |
| KU051710 | pIMP-FJ1503 | IMP-4 | Citrobacter freundii | 50546 |
| KU295134 | pBK32602 | KPC-3 | Escherichia coli | 87982 |
| KU726588 | pIMP-DS1516 | IMP-4 | Escherichia coli | 54449 |
| KU862632 | pIMP-KP1495 | IMP-4 | Klebsiella Pneumoniae | 51591 |
| KU886034 | pIMP-HZ1 | IMP-4 | Klebsiella Pneumoniae | 51599 |
| KX711879 | P378-IMP | IMP-4 | Pseudomonas aeruginosa | 51207 |
| KX711880 | p1220-IMP | IMP-4 | Klebsiella Pneumoniae | 46629 |
| KX928750 | pCRKP-1-KPC | KPC-1 | Klebsiella Pneumoniae | 65595 |
| KX928751 | pCRKP-5-KPC | KPC-1 | Klebsiella Pneumoniae | 68482 |
| KY128483 | pKm38_N | KPC-1 | Klebsiella oxytoca | 68763 |
| KY128484 | pKm38_N_2 | KPC-2 | Klebsiella michiganensis | 69888 |
| KY271413 | pKL49 | KPC-1 | Klebsiella Pneumoniae | 54345 |
| KY913900 | p4-IPM | IMP-4 | Klebsiella oxytoca | 61680 |
| MF072962 | pP10159-2 | IMP-4 | Citrobacter freundii | 51104 |
| MF344557 | p10677-IMP | IMP-4 | Klebsiella Pneumoniae | 56893 |
| MF344559 | p128379-IMP | IMP-4 | Enterobacter hormaechei | 42279 |
| MG764553 | pA3295-KPC | KPC-1 | Klebsiella Pneumoniae | 153274 |
| MH128095 | pTBCZNDM02 | NDM-1 | Klebsiella Pneumoniae | 36736 |
| MH727565 | pIMP-Enterobacter cloacae14-57 | IMP-4 | Citrobacter freundii | 51795 |
| CP024879 | pNH25.5 | NDM-1 | Klebsiella Pneumoniae | 38383 |
| JF785549 | p271A | NDM-1 | Escherichia coli | 35947 |
| JQ349086 | pTR3 | NDM-1 | Klebsiella Pneumoniae | 41187 |
| KJ413946 | pNDM-ECS01 | NDM-1 | Escherichia coli | 41190 |
| KT345947 | p0801-IMP | IMP-1 | Klebsiella pneumoniae | 42580 |
| KX784502 | p7121-IMP | IMP-1 | Klebsiella oxytoca | 42461 |
| KX784503 | p17285-IMP | IMP-8 | Citrobacter freundii | 43797 |
| KP900017 | pYNKP001-NDM | NDM-1 | Raoultella ornithinolytica | 41190 |
| HM126016 | pKOX105 | VIM-1 | Klebsiella oxytoca | 54641 |
| KC788405 | pKPC-LKEc | KPC-1 | Escherichia coli | 145401 |
| KF914891 | pECN580 | KPC-1 | Escherichia coli | 64935 |
| KR091915 | pKPC-DK05 | KPC-2 | Klebsiella Pneumoniae | 56728 |
